# Androgen Deprivation Therapy for Prostatic Cancer in Patients With Torsades de Pointes

**DOI:** 10.3389/fphar.2020.00684

**Published:** 2020-05-13

**Authors:** Pietro Enea Lazzerini, Iacopo Bertolozzi, Maurizio Acampa, Silvia Cantara, Maria Grazia Castagna, Laura Pieragnoli, Antonio D’Errico, Marco Rossi, Stefania Bisogno, Nabil El-Sherif, Mohamed Boutjdir, Franco Laghi-Pasini, Pier Leopoldo Capecchi

**Affiliations:** ^1^Department of Medical Sciences, Surgery and Neurosciences, University of Siena, Siena, Italy; ^2^Cardiology Intensive Therapy Unit, Department of Internal Medicine, Nuovo Ospedale San Giovanni di Dio, Florence, Italy; ^3^Stroke Unit, University Hospital of Siena, Siena, Italy; ^4^Department of Medical Sciences, Surgery and Neurosciences, Tuscan Centre of Pharmacovigilance, Florence, Italy; ^5^VA New York Harbor Healthcare System, SUNY Downstate Medical Center, New York, NY, United States; ^6^Division of Cardiology, Department of Medicine, NYU School of Medicine, New York, NY, United States; ^7^Retired, Siena, Italy

**Keywords:** Torsades de Pointes, androgen deprivation therapy, prostatic cancer, testosterone, sudden death

## Abstract

**Background:**

Men normally have shorter heart rate-corrected QT interval (QTc) than women, at least in part due to accelerating effects of testosterone on ventricular repolarization. Accumulating data suggest that androgen-deprivation therapy (ADT) used for the treatment of prostatic cancer, may increase Torsades de Pointes (TdP) risk by prolonging QTc. However, the evidence for such an association is currently limited to few case reports, in most cases deriving from the analysis of uncontrolled sources such as pharmacovigilance databases.

**Objective:**

To better determine the clinical impact of ADT on TdP development, we examined the prevalence of this therapy in a consecutive cohort of 66 TdP patients, prospectively collected over a ~10 years period.

**Methods and Results:**

We found and described four patients who were under ADT for prostatic cancer when TdP occurred, and in two cases degenerated to cardiac arrest. Notably, in this unselected population, ADTs unexpectedly represented the second most frequently administered QT-prolonging medication in males (4/24, 17%), after amiodarone. Moreover, in the ADT patients, a blood withdrawal was performed within 24 h from TdP/marked QTc prolongation occurrence and circulating concentration of androgens and gonadothropins were measured. As expected, all cases showed markedly reduced testosterone levels (total, free, and available).

**Conclusion:**

We provide evidence that a significant proportion of patients developing TdP were under treatment with ADT for prostatic cancer, thus confirming the clinical relevance of previous pharmacovigilance signals. An accurate assessment of the arrhythmic risk profile should be included in the standard of care of prostatic cancer patients before starting ADT.

## Introduction

Torsades de Pointes (TdP) is a polymorphic ventricular tachycardia occurring in patients with congenital or acquired long-QT syndrome (LQTS), which can degenerate to ventricular fibrillation (VF) and sudden cardiac death (SCD) (Drew et al., 2010).

Congenital LQTS results from genetically determined channelopathies (*inherited channelopathies*) directly or indirectly affecting the function of specific potassium (loss of function), sodium, or calcium (gain of function) channels critically involved in regulating ventricular action potential duration (APD) (El-Sherif et al., 2017). Although mutations of 17 different genes have been currently identified in clinically diagnosed LQTS, > 90% of genotype-positive cases involve three genes only, i.e. *KCNQ1* (encoding K_v_7.1 channel α-subunit, conducting the slow delayed rectifier potassium current I_Ks_; LQT1), *KCNH2* [Kv11.1 (also named hERG, human ether-à-go-go-related gene K^+^-channel) conducting the rapid delayed rectifier potassium current I_Kr_; LQT2], and SCN5A (Na_v_1.5, conducting the sodium current I_Na_; LQT3) (El-Sherif et al., 2017).

While congenital LQTS is relatively rare, occurring in ~1:2,000 live births, acquired LQTS is significantly more common, affecting up to 25–30% of hospitalized patients (Drew et al., 2010; El-Sherif et al., 2017; El-Sherif et al., 2018). Drugs, mainly hERG-potassium channel blockers, and electrolyte imbalances (hypokalemia, hypocalcaemia, hypomagnesemia) represent the most frequent causes of acquired LQTS (Drew et al., 2010; El-Sherif et al., 2018). Other important causes include structural heart diseases, bradyarrhythmias, endocrine disorders, liver diseases, nervous system injuries, HIV infection, starvation, hypothermia, and toxins (Drew et al., 2010; El-Sherif et al., 2018), as well as the recently identified “non-conventional” factors such as *autoimmune* (anti-Sjogren syndrome-A, anti-Ro/SSA antibodies) and *inflammatory cardiac channelopathies* (pro-inflammatory cytokines) (Lazzerini et al., 2017a; Lazzerini et al., 2017b; El-Sherif et al., 2018; Lazzerini et al., 2018; Lazzerini et al., 2019).

Although LQTS is traditionally classified as congenital or acquired, in most cases TdP results from an interaction of multiple factors operating concomitantly in the single patient (*multi-hit theory*) (Lazzerini et al., 2018). Accordingly, patients developing TdP present many simultaneous acquired risk factors (Drew et al., 2010), on average >4 (Lazzerini et al., 2017c). In addition, a recent study provided evidence that ~30% of individuals developing acquired LQTS are carriers of LQTS-related gene mutations, those on *KCNH2* being more frequent (Itoh et al., 2016). Moreover, specific common polymorphisms in LQTS related genes like KCNH2-K897T could also act as arrhythmic risk modifiers in association with other environmental risk factors/acquired factors reducing the repolarization reserve (Crotti et al., 2005).

In general, women have longer heart rate-corrected QT interval (QTc), and are at significantly greater risk for drug-induced TdP, than men (Salem et al., 2016). Gender difference in LQTS/TdP risk likely reflects, at least partly, direct influences of sex hormones on cardiac electrophysiology. For example, estradiol prolongs, while progesterone and testosterone shorten QTc (Salem et al., 2016).

Preclinical studies show that testosterone can both increase the repolarizing potassium currents I_Kr_ and I_Ks_, and decrease the depolarizing L-type calcium current, I_CaL_ (Salem et al., 2016). Recently, in seven male patients with TdP, hypogonadism was diagnosed in all cases, and after correction for low testosterone levels, QTc shortened with no TdP recurrence (Salem et al., 2018). Moreover, by analyzing international pharmacovigilance databases, the same authors found that androgen-deprivation therapy (ADT) was associated with higher reporting odds-ratios for drug-induced-LQTS/TdP when compared to testosterone-replacement therapy (Salem et al., 2018; Salem et al., 2019a; Salem et al., 2019b). Although interesting, these pharmacovigilance data are limited as derived from uncontrolled sources. Thus, to better determine the actual clinical impact of ADT on TdP development, we examined the prevalence of this therapy in a cohort of consecutive patients experiencing TdP, over a ~10 years period.

## Methods

### Study Population

Local ethics committee approved the study, and patients gave their oral and written informed consent in accordance with the Principles of the Declaration of Helsinki.

Since 2008, we have been prospectively enrolling patients who experienced TdP, independent of on-going therapies and concomitant diseases. To date (December 2019), the cohort consists of 66 patients, including 42 females and 24 males. All patients were admitted in the Cardiology Intensive Therapy Unit. Demographic, clinical and laboratory characteristics of study patients, as well as on-going treatment with QTc prolonging medications are provided in the **Table 1**.

**Table 1:** Demographic, clinical, and laboratory characteristics of patients with Torsades de Pointes.

Patients, n	66
Age, median years (range)	81 (30–95)
Males, n	24/66 (36%)
Age, years (range)	78.5 (35–91)
Family history of sudden cardiac death	0/8
Mean QTc, ms (range)	593.7 ± 79.2 (490–910)
Heart rate, ppm (range)	68.0 ± 21.5 (30–130)
Mean QTc-prolonging risk factor number per patient^§^	5.1 ± 1.7
Electrolyte imbalances, n	49/65 (75%)
Hypokaliemia (<3.5 mEq/L)	34/61 (56%)
Hypocalcemia (<8.0 mg/dl)	23/51 (45%)
Hypomagnesemia (<1.5 mg/dl)	7/36 (19%)
Concomitant diseases*, n	61/66 (92%)
*Cardiac diseases*	56/66 (85%)
Left ventricular hypertrophy	29/66 (44%)
Dilated cardiomyopathy/heart failure	21/66 (32%)
II-III degree atrioventricular block	18/66 (27%)
Acute coronary syndrome	12/66 (18%)
Chronic coronary artery disease	9/66 (14%)
Sinus bradycardia	
<60 ppm	8/66 (12%)
<50 ppm	6/66 (9%)
*Extra-cardiac diseases*	27/66 (41%)
Diabetes mellitus type II	19/66 (29%)
Chronic kidney disease	12/66 (18%)
Hypothyroidism	2/66 (3%)
Anorexia nervosa/starvation	2/66 (3%)
Anti-Ro/SSA positivity , n	21/38 (55%)
Systemic inflammation, n^†^	55/66 (83%)
C-reactive protein, mg/dl (range)	2.66 (0.1–29.65)
QTc prolonging-medications, n	50/66 (76%)
Amiodarone	16/66 (24%)
Trazodone	6/66 (9%)
Citalopram	5/66 (8%)
Sertraline	5/66 (8%)
**Androgen deprivation therapy**	**4/66 (6%)**
Fluconazole	3/66 (5%)
Levofloxacin	3/66 (5%)
Quetiapine	3/66 (5%)
Mean medication number per patient	1.2 ± .0.9
QTc prolonging-medications in males, n	17/24 (71%)
Amiodarone	5/24 (21%)
**Androgen deprivation therapy**	**4/24 (17%)**
Trazodone	3/24 (12%)
Escitalopram	1/24 (4%)
Sertraline	1/24 (4%)
Azytromycin	1/24 (4%)
Fluconazole	1/24 (4%)
Promazine	1/24 (4%)
Mean medication number per patient	1.1 ± .0.9

Except where indicated otherwise, data are expressed as mean ± standard deviation or median (range).

^§^Including electrolyte imbalances, diseases, anti-Ro/SSA positivity, systemic inflammation, and QTc-prolonging medications.

*Diseases recognized to be a risk factor for QTc prolongation.

^†^Increased C-reactive protein level (>0.5 mg/dl) with or without a definite inflammatory disease.

The ECG used for the QTc interval calculation is the one with the longest available QTc value before the TdP event.

Family history of SCD, i.e. occurring in a first-degree family member before the age of 40, was systematically searched in eight patients only, including the four patients under ADT plus the four subjects in whom TdP occurred before the age of 50.

Chronic kidney disease was defined by an estimated glomerular filtration rate (eGFR) < 60 mL/min/1.73 m^2^.

Serum potassium, calcium or magnesium measurements available before replacement therapy in 61, 51, and 36 out of 66 patients, respectively; anti-Ro/SSA antibodies tested in 38 out of 66 patients.

QTc, corrected QT.

Anti-Ro/SSA, anti-Sjogren syndrome-A antibodies.

Androgen deprivation therapy frequency and percentages are reported in bold.

QTc interval was calculated by using the Bazett’s formula (dividing the QT interval by the square root of the R-R interval). The ECG used for the QTc interval calculation is the one with the longest available QTc value before the TdP event. The presence of a family history of SCD, i.e. occurring in a first-degree family member before the age of 40, was not systematically searched in the overall TdP cohort, given the high mean age of patients (~80 years).

Specifically regarding QT-prolonging drugs, we evaluated whether TdP occurred when patients were under ADT, including gonadotrophin-releasing hormone-receptor agonists/antagonists (leuprolide, buserelin, goserelin, triptorelin/degarelix, abarelix), cytochrome-17 inhibitor (abiraterone), nonsteroidal androgen-receptor antagonists (bicalutamide, flutamide, nilutamide, enzalutamide), and 5α-reductase inhibitors (finasteride, dutasteride).

### Sex Hormones Measurement

In order to demonstrate that in TdP patients under ADT a condition of hypogonadism was effectively present, in these subjects a blood sample was obtained within 24 h from TdP/marked QTc prolongation occurrence and levels of testosterone (total, free, and bioavailable), sex-hormone binding protein (SHBG), follicle stimulating hormone (FSH), luteinizing hormone (LH), dehydroepiandrosterone sulphate (DHEA-S), and androstenedione were assessed. All measurements were performed by an automatic chemiluminescent immunoassay system. Testosterone (total), LH, and FSH were measured by UniCel DxI 800 (Beckman Coulter), while SHBG, DHEA-S, and androstenedione levels by Immulite 2000 (Siemens). Free and bioavailable testosterone were calculated based on SHBG and total testosterone concentration, considering albumin as a constant (average albumin concentration of 4.3 g/dl). This parameter more accurately reflects the level of bioactive testosterone than does the sole measurement of total serum testosterone. Testosterone circulates in plasma unbound (free approximately 2–3%), bound to specific plasma proteins (SHBG), and weakly bound to nonspecific proteins such as albumin. The SHBG-bound fraction is biologically inactive, because of the high binding affinity of SHBG for testosterone. Free testosterone measures the free fraction; bioavailable testosterone includes free plus weakly bound to albumin.

## Results

The patients in the whole TdP cohort showed a high prevalence of recognized acquired QT-prolonging risk factors, on average five per patient, including those “conventional”, mainly organic heart diseases (85%), QT-prolonging medications (76%), and electrolyte imbalances (75%), and “non-conventional”, such as systemic inflammation (83%) and anti-Ro/SSA autoantibodies (55%).

Four out of 66 patients (6%) were taking ADTs for prostatic cancer, 2 leuprolide and 2 bicalutamide, unexpectedly representing the fifth most frequently used QT-prolonging medication in the overall cohort. Notably, ADTs were the second most frequently administered medications in males (17%) after amiodarone (**Table 1**). It should also be noted that this percentage was ~7-times higher than that observed in a control population of 123 elderly males comparable for age (median 77.0 years), consecutively admitted in our Internal Medicine Unit at the University Hospital of Siena, during the last 2 years within the ASTEROID study [2.4%, 3 patients under ADT (2 leuprolide, 1 abiraterone) for prostatic cancer; relative risk 6.83, 95% confidence interval 1.63–28.61; two-sided Fisher’s exact test: p=0.014].

**Figure 1:**
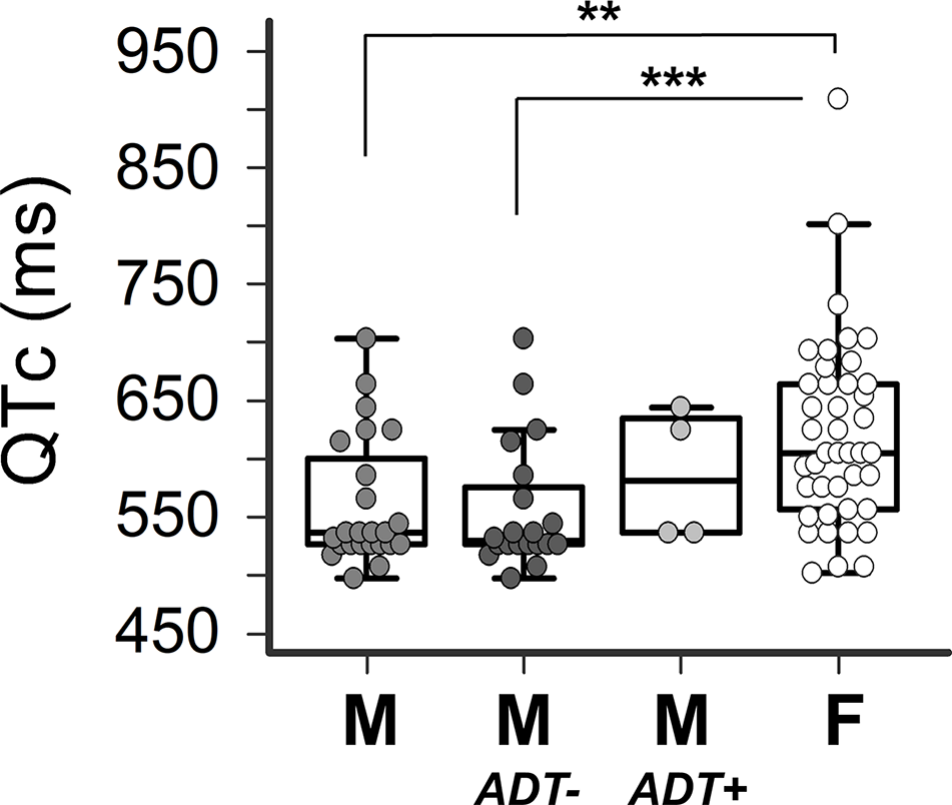
Impact of sex and androgen deprivation therapy on QTc in TdP patients. Comparisons of QTc in males (M, n=24), males untreated with androgen deprivation therapy (M-ADT-, n=20), males treated with androgen deprivation therapy (M-ADT+, n=4), and females (F, n=42). ***p < 0.001, **p < 0.01; M-ADT+ *vs* F: p=0.38; M-ADT- *vs* M-ADT+: p=0.14. All comparisons were performed by the two-tails Mann-Whitney test: QTc: heart-rate corrected QT interval; TdP: Torsades de pointes.

As expected, in the overall cohort the mean QTc was longer in females than males (615.9 ± 82.4 *vs* 554.7 ± 56.3 ms, p=0.007), despite the absence of significant differences in terms of age and number of QT-prolonging risk factors per patient (**Table 2**; **Figure 1**). Notably, such a difference disappeared when females were compared to males treated with ADT (580.0 ± 58.3 ms, p=0.38). Conversely, the difference became more evident when the comparison was with male patients without ADT (549.6 ± 56.0 ms, p=0.0005). Thus, the mean QTc values tended to be higher in ADT-treated *vs* –untreated males, although not significantly (p=0.14) given the limited sample size (**Table 2**; **Figure 1**).

**Table 2:** Demography, QTc and QT-prolonging risk factors in patients with TdP: comparisons by sex and androgen deprivation therapy (ADT).

	Males	Males without ADT	Males with ADT	Females
n	24	20	4	42
Age, years (median [range])*	78.5 [35–91]	78 [35–91]	84 [67–90]	82 [30–95]
QTc, ms (mean ± SD [range])*	556.4 ± 56.1 [490–700]	549.6 ± 56.0 [490–700]	580.0 ± 58.3 [530–640]	615.9 ± 82.4 [495–910]
Heart rate, bpm (mean ± SD [range])	71.1 ± 21.3 [40–120]	73.5 ± 21.6 [40–120]	59.2 ± 17.0 [40–80]	68.3 ± 21.5 [30–130]
QT-prolonging risk factor number per patient^§^ (mean ± SD)*	5.5 ± 1.9	5.6 ± 2.0	5.0 ± 0.8	4.9 ± 1.6

TdP, Torsades de Pointes; SD, standard deviation; ADT, androgen deprivation therapy; bpm, beats per minute.

*No significant differences for all comparisons (two-tails Mann-Whitney test).

^§^Including electrolyte imbalances, diseases, anti-Ro/SSA positivity, systemic inflammation, and QTc-prolonging medications.

Family history of SCD was systematically searched in eight patients only, i.e. the four patients under ADT plus the four subjects in whom TdP occurred before the age of 50, but in all cases it was negative (**Table 1**).

A detailed description of the four patients developing TdP under ADT is provided below and in the **Table 3**.

**Table 3:** Demographic, clinical, and laboratory characteristics of patients by case.

	Patient 1	Patient 2	Patient 3	Patient 4
Age (years)	90	67	83	84
QTc (ms)	530	620	530*	640
TdP	Yes	Yes	Yes	Yes
Cardiac arrest/sudden death	No	Yes	Yes	No
Androgen deprivation therapy	Leuprolide	Leuprolide	Bicalutamide	Bicalutamide
Prostatic cancer	Yes	Yes	Yes	Yes
***Concomitant QT-prolonging risk factors***				
Pharmacologic	–	–	Amiodarone	Amiodarone
Non-pharmacologic	Acute complete AVB, heart failure, LVH, elevated CRP	LVH, sinus bradycardia, ipokaliemia	Chronic coronary artery disease, DCM, chronic kidney disease, hypocalcemia, elevated CRP (UTI)	ACS, heart failure, LVH, elevated CRP (sepsis)
***Hormonal profile***				
Total testosterone (r.v. 2.7–10.9 ng/ml)	**0.18 ↓**	**<0.1 ↓**	**0.65 ↓**	**0.28 ↓**
Sex hormone binding globulin (SHBG) (r.v. 10–57 nmol/L)	56.1	**64.1↑**	**101.0↑**	**90.6↑**
Free testosterone (r.v. 0.06–2.2 ng/ml)	**<0.005 ↓**	**<0.005 ↓**	**<0.005 ↓**	**<0.005 ↓**
Bioavailable testosterone (r.v. 0.15–5.7 ng/ml)	**0.05 ↓**	**<0.05 ↓**	**0.12 ↓**	**0.06 ↓**
Luteinizing hormone (LH) (r.v. 0.8–8 mU/ml)	**<0.2 ↓**	**<0.2 ↓**	**10.5 ↑**	**<0.2 ↓**
Follicle stimulating hormone (FSH) (r.v. 0.7–11 mU/ml)	3.9	6.0	**14.4 ↑**	4.0
Androstenedione (r.v. 0.4–3.1 ng/ml)	1.95	**3.43↑**	1.93	1.33
Dehydroepiandrosterone-sulphate (DHEA-S) (r.v. 120–520 µg/dl)	**<15 ↓**	**<15 ↓**	**20.7 ↓**	**33.9 ↓**

TdP, torsades de pointes; AVB, atrio-ventricular block; LVH, left ventricular hypertrophy; DCM, dilated cardiomyopathy; UTI, urinary tract infection; ACS, acute coronary syndrome; CRP, C-reactive protein; r.v., reference values.

*Value measured during biventricular paced rhythm.

Out of reference values are reported in bold, while arrows indicate increase (up arrows)/decrease (down arrows).

### Patient 1

**Figure 2:**
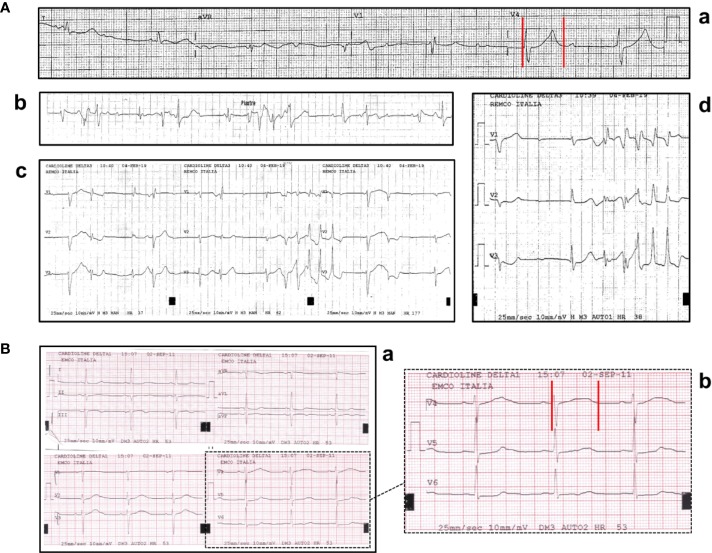
**(A)** ECG findings in patient 1: (a) QTc prolongation (530 ms); (b-d) recurrent episodes of self-terminating Torsades de Pointes. **(B)** ECG findings in patient 2: sinus bradycardia with marked QTc prolongation (610 ms). Red vertical lines in lead V4 show the QT interval.

A 90-year-old man presented to the emergency department with shortness of breath and fatigue for several days. The medical history included hypertension, hypercholesterolemia, hyperuriceamia, combined mitral and aortic valve stenosis, and prostatic cancer. He reported no cases of SCD among family members. Ongoing therapies included simvastatin, allopurinol, lansoprazole, silodosin, and the gonadotrophin-releasing hormone-receptor agonists/antagonists leuprolide (3.75 mg/mo for several years). On admission, electrocardiogram (ECG) showed third-degree atrioventricular block with low-rate ventricular escape (~40 beats per minute, bpm), QTc prolongation (~530 ms), and frequent self-terminating runs of TdP (**Figure 2A**), also confirmed by telemetry. Chest radiograph demonstrated interstitial pulmonary edema, while echocardiography showed left ventricular hypertrophy (LVH, with preserved ejection fraction), diastolic dysfunction, stenosis of mitral (moderate) and aortic (severe) valves with left atrial enlargement, pulmonary hypertension, and right ventricular dilatation. Blood tests showed increased levels of brain natriuretic peptide (BNP, 826 ng/L; r.v. < 100) and inflammatory markers (C-reactive protein, CRP, 3.7 mg/dl, r.v. < 0.5; fibrinogen 606 mg/dl, r.v. 200–400), in the absence of any evident site of infection; conversely, potassium, calcium, magnesium, and creatinine levels were normal. Tests for circulating anti-Ro/SSA antibodies were negative. The patient was treated with intravenous diuretics, and a VDD-R pacemaker (PM) was implanted, resulting in a rapid improvement of dyspnea and fatigue. In the following days, clinical conditions completely normalized, and ECG monitoring demonstrated no recurrence of ventricular arrhythmias, specifically TdP, in the presence of a proper PM activation (65 bpm). The patient continued ADT, and after 1 year of follow-up no TdP recurrences were seen.

### Patient 2

A 67-year-old man presented an out-of-hospital episode of syncope during nighttime with convulsive seizures. The history included hypertension and a recently diagnosed prostatic cancer, under treatment with irbesartan, hydrochlorothiazide, and the gonadotrophin-releasing hormone-receptor agonists/antagonists leuprolide (depot formulation: 22.5 mg every 3 months, for several months). He had no family history for SCD. The ECG on admission demonstrated sinus bradycardia and marked QTc prolongation (~620 ms) (**Figure 2B**). In the following days, ECG monitoring did not reveal repetitive ventricular arrhythmias, but only isolated, monomorphic ventricular ectopic beats; conversely, persistent sinus bradycardia was present, particularly evident during night-time. Laboratory investigation was remarkable for low potassium levels (2.8 mEq/l), while other tests for electrolytes, inflammatory markers, and anti-Ro/SSA antibodies were normal/negative. Echocardiography demonstrated LVH with normal systolic function, left atrial enlargement, and moderate mitral valve regurgitation, in the absence of mitral valve prolapse and/or mitral annular disjunction. Neurological investigations, including brain computed tomography and magnetic resonance imaging, and electroencephalography were unremarkable. The patient was treated with intravenous potassium chloride, and hydrochlorothiazide was stopped; however, a less evident but significant QTc prolongation (>500 ms) persisted also after potassium levels normalization. Therefore, lueuprolide was also (temporarily) stopped, although its potential QT-prolonging effects were supposed to be long-lasting due to the depot formulation. In order to verify whether clinical manifestation were due to LQTS-induced ventricular arrhythmias, before the discharge a loop-recorder was positioned. Three months later the patient developed an out-of-hospital cardiac arrest and suddenly died during nighttime: the implanted device recorded a polymorphic ventricular tachycardia (TdP) rapidly degenerated to VF (unfortunately, the printed trace of the arrhythmic episode from the loop recorder is not available).

### Patient 3

**Figure 3:**
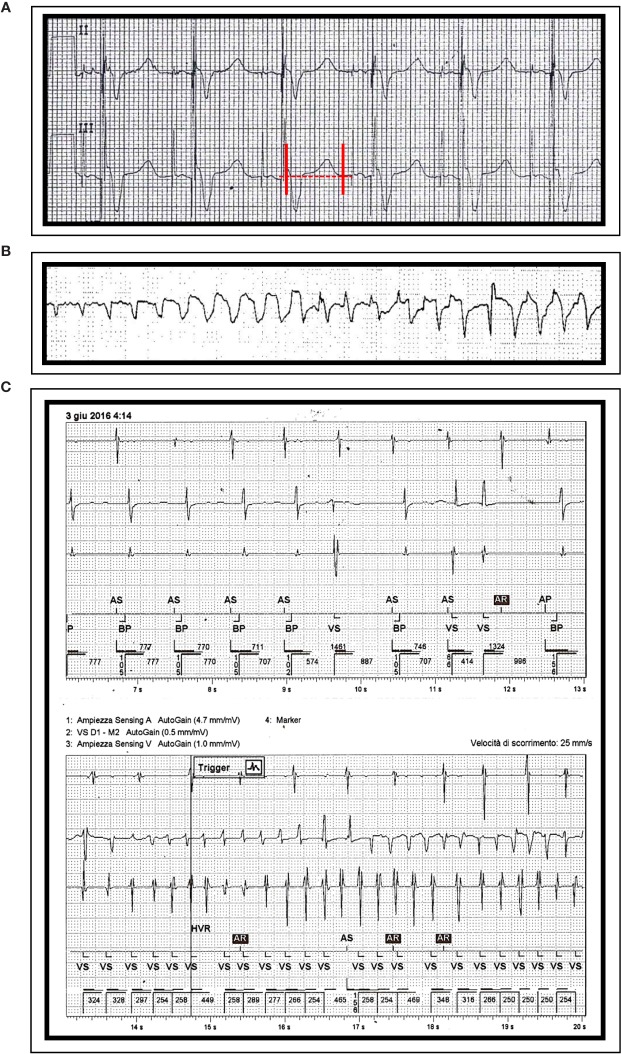
ECG findings in patient 3: **(A)** QTc prolongation (530 ms) during paced rhythm (QRS 200 ms). Episodes of Torsades de Pointes, as recorded by ECG monitoring **(B)** and by interrogating the PM **(C)**. Red vertical lines in lead III show the QT interval.

A 83-year-old man was admitted for worsening dyspnea due to acute congestive heart failure. His medical history was remarkable for chronic coronary artery disease, dilated cardiomyopathy with biventricular PM implantation for cardiac resynchronization therapy, chronic kidney disease (mild), and metastatic prostatic cancer. The patient had no history of SCD among family members. Current medications included aspirin, clopidogrel, furosemide, ivabradine, atorvastatin, allopurinol, the (potential) QT-prolonging drug amiodarone, and the nonsteroidal androgen-receptor antagonists bicalutamide (150 mg/d for several years). On admission, ECG showed PM-induced rhythm (75 bpm) and QTc, although sub-optimally measured on a ventricular paced beat, was prolonged (~530 ms) (**Figure 3A**), while echocardiography revealed left ventricular dilation, with diffuse contraction abnormalities resulting in a severe reduction of the global ejection fraction (~25–30%). Blood tests were significant for increased BNP (1,002 ng/L) and mild CRP level elevation (2.7 mg/dl), while troponin I was in the normal range. Anti-Ro/SSA antibodies were absent. The patient was treated with high-dose diuretics and inotropic agents (dopamine and dobutamine), resulting in an early but significant improvement of dyspnea. However, few days later, the patient suddenly presented repeated episodes of TdP (**Figures 3B, C**) rapidly degenerating to VF and cardiac arrest, requiring multiple DC-shocks. Laboratory analysis showed mild hypocalcaemia (4.3 mg/dl; r.v. 4.4–5.2 mg/dl; potassium and magnesium levels in range). Intravenous magnesium sulfate and calcium gluconate were started. Moreover, inotropic agents were stopped, and the PM was reprogrammed (lower stimulation rate 80 ppm). However, two further TdP episodes occurred in the following 2 h. At that time, the patient presented chills and dysuria, with marked increase of inflammatory markers (CRP 16.5 mg/dl; procalcitonin 1.46 ng/ml, r.v. < 0.5), probably due to acute urinary tract infection in a catheterized patient, and antibiotic therapy (amoxicillin/clavulanate) was started. The electric storm ceased, and the clinical conditions of the patient markedly improved.

In the next days, laboratory abnormalities progressively normalized and no further arrhythmic events were demonstrated, nor at ECG monitoring or when interrogating the PM later on. ADT was not stopped after discharge. However, no information on possible TdP recurrences is available as the patient was lost early on to follow-up.

### Patient 4

**Figure 4:**
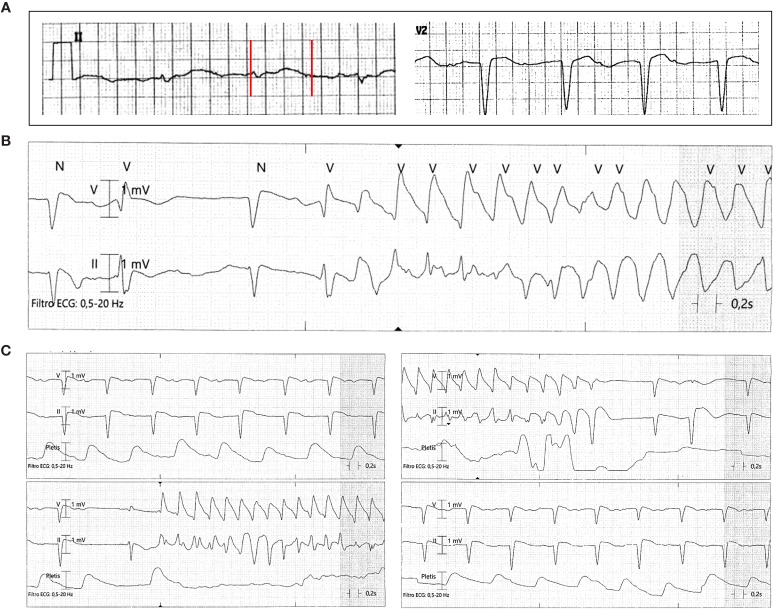
ECG findings in patient 4: **(A)** QTc prolongation (640 ms); **(B**, **C)** recurrent episodes of nonsustained Torsades de Pointes. Red vertical lines in lead II show the QT interval.

A 84-year-old man presented to the emergency department for syncope. Medical history included hypertension, hypercholesterolemia, hyperuricemia, heart failure, recurrent atrial flutter/fibrillation, and prostatic cancer, under treatment with valsartan, hydrochlorothiazide, doxazosin, lercanidipine, simvastatin, febuxostat, furosemide, warfarin, pantoprazole, the (potential) QT-prolonging drug amiodarone, and the nonsteroidal androgen-receptor antagonists bicalutamide (50 mg/d for several years). He had no family history of SCD. The ECG on admission showed first-degree atrioventricular block (PR ~500 ms), complete left bundle branch block (QRS ~140 ms), and marked QTc prolongation (~640 ms) (**Figure 4A**). Laboratory assessment revealed increase in troponin I (2.31 ng/ml, r.v. < 0.07) and inflammatory markers (CRP 14.6 mg/dl; procalcitonin 3.48 ng/ml), while electrolytes (potassium, calcium, magnesium), and anti-Ro/SSA antibodies were normal/negative. A chest X-ray demonstrated a right basal lung consolidation, and blood cultures were positive for *Escherichia coli*. Echocardiography showed LVH with left ventricular dysfunction (ejection fraction, ~40%), while a brain computed tomography was unremarkable. The patient was treated with diuretics and antibiotics (ceftriaxone) with an initial improvement of the clinical conditions. However, during nighttime, recurrent episodes of nonsustained TdP occurred (**Figures 4B, C**), and intravenous magnesium sulfate was started. A coronary angiography revealed multi-vessels coronary artery disease, with sub-occlusion of the middle tract of the right coronary artery. The lesion was treated by percutaneous angioplasty and stenting. In the next days, inflammatory markers and troponin levels progressively normalized, and no ventricular arrhythmias recurred. After discharge ADT was continued, but the patient was lost in the follow-up.

### Sex Hormones Measurement

In all the four TdP cases presented, testosterone levels (total, free, and available) were markedly reduced when TdP occurred. As expected, concentrations were particularly low in patients under treatment with leuprolide (patients 1 and 2), in whom also LH was suppressed. Conversely, in the patient 3, FSH and LH levels were increased, as a result of bicalutamide interference on the gonadotrophins negative feedback. Finally, patient 4, despite bicalutamide treatment, additionally showed LH suppression. This finding, further contributing to low testosterone levels, was interpreted as probably due to sepsis-induced functional central hypogonadism. Noteworthy, in all cases DHEA-S levels were significantly reduced, while androstenedione concentrations were in the normal range (**Table 3**).

## Discussion

Our results provide evidence *for the first time* that a significant proportion of patients developing TdP (~15% of males) were under treatment with ADT for prostatic cancer, thus confirming the clinical relevance of previous pharmacovigilance signals (Salem et al., 2018; Salem et al., 2019a; Salem et al., 2019b). These data further support an important physiological role for testosterone in preserving ventricular repolarization in males (Salem et al., 2016), and emphasize the clinical impact of ADT-induced hypogonadism in promoting TdP, particularly in the presence of other concomitant QT-prolonging risk factors.

Increasing evidence in the recent years supports the hypothesis that ADT can increase the risk of life-threatening arrhythmias, specifically TdP due to QTc prolongation (Barber et al., 2019). Clinical interventional studies consistently demonstrated that in men with prostate cancer, ADT is associated with a significant ~10–20 ms QTc lengthening (Smith et al., 2010; Tolcher et al., 2012; Gagliano-Jucá et al., 2018). Moreover, a very recent translational study by Salem et al. showed that the androgen receptor antagonist enzalutamide significantly prolongs APD in induced pluripotent stem cells-derived cardiomyocytes, also promoting after depolarizations and triggered activity. These effects were associated to I_Kr_ inhibition and I_Na_ enhancement, and were reversed by dihydrotestosterone (Salem et al., 2019a). Nevertheless, the evidence of an association between ADT and TdP/SCD is currently limited to single case reports, in most cases deriving from the analysis of uncontrolled sources such as pharmacovigilance databases (Salem et al., 2019b). In fact, to date only three published cases of TdP occurring in prostatic cancer patients under ADT can be found in PubMed-Medline, two with abiraterone and one with the combined treatment leuprolide/bicaluatimde (Rodieux et al., 2015; Khan and Kneale, 2016; Hasegawa et al., 2019).

In an effort to fill this knowledge gap, we here analyzed a large prospective cohort of consecutive, unselected patients with marked QTc prolongation complicated with TdP, and demonstrated that ongoing ADT was an unexpectedly common finding. In fact, in male patients (and even in the overall cohort), ADT was more frequently used than a number of well-recognized QT-prolonging drugs, such as antidepressants, neuroleptics, or antimicrobials. Notably, such a high frequency is not merely due to an increased prescription of these drugs in our geographic area, as indicated by the significantly lower prevalence observed in a control population of elderly males who recently were consecutively admitted to our unit.

By providing support to the preliminary literature data, both basic and clinical, our data emphasize how the alertness to the potential arrhythmic hazard of this class of drugs should be greater than currently. In fact, it is important to underline how most of ADTs, such as bicalutamide, are not at the moment included in the website crediblemeds.org as risk factors for LQTS/TdP. For example, leuprolide and degarelix are listed, but only as drugs carrying a *possible risk* of TdP, (i.e. potentially causing QTc prolongation, but currently lacking evidence for a risk of TdP when taken as recommended) (Barber et al., 2019). Notably, in our patients, TdP occurred despite ADTs were administered according to the current recommendations.

At the same time, our data also indicate that ADT alone cannot account for TdP occurrence in any of our four patients. Indeed, this is also true for all known QT-prolonging risk factors, either conventional or non-conventional, when considered separately (Lazzerini et al., 2018). In accordance with the multi-hit theory (Lazzerini et al., 2018), these drugs may have contributed to TdP development in our patients by acting in a synergistic manner with the other concomitant QT-prolonging factors, more frequently conventional such as structural heart disease and electrolyte imbalances, but also non-conventional factor such as systemic inflammation. Specifically, patient 1 presented with heart failure and LVH, and TdP occurred during acute complete atrioventricular-block, in the presence of high CRP. Patient 2 had hypertensive LVH and developed TdP in the presence of sinus bradycardia and hypokalemia. Patient 3 was affected by chronic coronary artery disease, dilated cardiomyopathy, and chronic kidney disease; when TdP occurred, he was taking amiodarone, and CRP was elevated due to acute urinary infection. Additionally, in this patient neuro-humoral activation due to acute congestive heart failure and intravenous inotropic agent administration may have also significantly contributed to the electrical storm. In patient 4, who had heart failure, LVH, and was chronically treated with amiodarone, TdP developed in a composite setting of systemic inflammatory activation due to pneumonia-derived sepsis, and acute coronary syndrome, all probably contributing to the arrhythmic event. In agreement with these findings, in most of the previously reported cases of ADT-associated TdP, both published and pharmacovigilance databases-derived, multiple QT-prolonging risk factors, were concomitantly present in the same patient (Salem et al., 2019b). Thus, in these cases as well as in our patients, delineating the specific contribution of ADT to TdP development remains to be seen.

From a pathophysiological point of view, drug-induced hypogonadism seems to be the key mechanism enhancing TdP risk in ADT patients. In particular, by removing the physiological shortening effects of testosterone on cardiac APD, ADT can reduce the repolarization reserve, thereby increasing the susceptibility to develop significant QTc prolongation, even life-threatening TdP. Indeed, all the four cases described in the present study showed a marked reduction of circulating testosterone, with negligible levels of the free fraction of the hormone, i.e. the biologically active form responsible for the electrophysiological effects on the cardiomyocyte. This is valid also for patient 2, although the loop recorder revealed that TdP occurred 3 months after the last administration of ADT, as the drug depot formulation maintained him also at that time at castrated testosterone levels (i.e. ≤0.5 ng/ml) (Chu et al., 2002). Accordingly, ADT-treated patients showed a mean QTc which was comparable to females and longer than ADT-untreated males, although no significant differences in terms of QT-prolonging risk factors load was observed among sub-groups.

Notably, a significant decrease of DHEA-S is also observed in all patients. Although no clinical or basic data are currently available regarding a possible specific impact of this hormone on ventricular repolarization, it cannot be ruled out. In fact, a link between DHEA-S and other arrhythmic disorders, specifically atrial fibrillation, has been reported already (Krijthe et al., 2014). Moreover, in Patient 3 bicalutamide administration was responsible for the concomitant increase of both FSH and LH gonadotrophins. Recent data indicate that FSH receptors are present in the myocardium (Salem et al., 2016), and FSH levels positively correlated with QTc in both genders (Abehsira et al., 2016). This evidence intriguingly points to direct electrophysiological effects of this gonadotrophin on the heart, potentially providing an additional QTc prolonging component specifically occurring in patients with hypergonadotropic hypogonadism (Salem et al., 2016). All these aspects warrant further investigation.

This study has some limitations. The sample size of male patients included is relatively small. However, it should be emphasized that TdP is not a frequent event (specifically in males), at least in part because it is frequently overlooked as it rapidly degenerate to VF and cardiac arrest (Drew et al., 2010). Another possible limitation is the lack of genetic data on potentially concomitant LQTS-associated mutations. In fact, previous studies demonstrated the presence of a subclinical congenital LQTS in up to ~20–30% of patients developing drug-induced TdP or more in general acquired LQTS (Drew et al., 2010; Itoh et al., 2016). In this regard, genetic testing would have been important to better define the total load of QT-prolonging risk factors actually involved in each patient. Nevertheless, the mean age of the 66 patients analyzed in this study is overall quite high and consistently higher as compared to that of patients described in studies assessing the genetic background (i.e. mutations, not common polymorphisms) of patients with acquired LQTS. A younger age (< 40 years) at presentation was found to be an independent predictor of harboring a pathogenic LQTS mutation among patients otherwise classified as having acquired LQTS (Itoh et al., 2016). This strengthens the idea that ADT might act as an acquired risk factor for QT prolongation and TdP risk, in addition to other hits and even in the absence of a strong genetic background (i.e. mutations).

## Conclusions

Our data suggest that the torsadogenic potential of ADTs is higher than expected, particularly in the presence of other recognized QT-prolonging factors. Since ADT is a cornerstone for the treatment of prostatic cancer, an accurate clinical assessment of the arrhythmic risk profile should be included in the standard for care of these patients.

## Data Availability Statement

The datasets generated for this study are available on request to the corresponding author.

## Ethics Statement

Written informed consent was obtained from the individuals for the publication of any potentially identifiable images or data included in this article.

## Author Contributions

Conception and design of the work: PL. Substantial contributions to the acquisition of data for the work: PL, IB, MA, SC, LP, AD’E, and SB. Substantial contributions to the analysis of data for the work: PL, IB, and PC. Substantial contributions to the interpretation of data for the work: PL, IB, MA, SC, FL-P, and PC. Drafting the work: PL, IB, AD’E, and PC. Revising the draft of the work critically for important intellectual content: IB, MA, SC, MC, LP, AD’E, MR, SB, and NE-S. MB, FL-P, and PC. Final approval of the version to be published: IB, MA, SC, MC, LP, AD’E, MR, SB, NE-S, MB, FL-P, and PC. Agreement to be accountable for all aspects of the work in ensuring that questions related to the accuracy or integrity of any part of the work are appropriately investigated and resolved: IB, MA, SC, MC, LP, AD’E, MR, SB, NE-S. MB, FL-P, and PC.

## Funding

This work was funded by: Ministero dell’Istruzione, dell’Università e della Ricerca (MIUR), Progetti di Rilevante Interesse Nazionale (PRIN), and Bando 2017, protocollo 2017XZMBYX; Italian Medicines Agency (AIFA), program for funding active pharmacovigilance projects, “Arrhythmia riSk associated to iatrogenic QT pRolongation in hOspitalIzed elDerly patients” (the ASTEROID study).

## Conflict of Interest

The authors declare that the research was conducted in the absence of any commercial or financial relationships that could be construed as a potential conflict of interest.
